# Transcriptional Regulation of ROS Homeostasis by the ERR Subfamily of Nuclear Receptors

**DOI:** 10.3390/antiox10030437

**Published:** 2021-03-12

**Authors:** Charlotte Scholtes, Vincent Giguère

**Affiliations:** 1 Rosalind and Morris Goodman Cancer Research Centre, McGill University, Montréal, QC H3A 1A3, Canada; charlotte.scholtes@mcgill.ca; 2 Department of Biochemistry, McGill University, Montréal, QC H3G 1Y6, Canada

**Keywords:** estrogen-related receptor, metabolism, mitochondria, nuclear receptors, redox signaling, transcription

## Abstract

Reactive oxygen species (ROS) such as superoxide anion (O_2_^•−^) and hydrogen peroxide (H_2_O_2_) are generated endogenously by processes such as mitochondrial oxidative phosphorylation, or they may arise from exogenous sources like bacterial invasion. ROS can be beneficial (oxidative eustress) as signaling molecules but also harmful (oxidative distress) to cells when ROS levels become unregulated in response to physiological, pathological or pharmacological insults. Indeed, abnormal ROS levels have been shown to contribute to the etiology of a wide variety of diseases. Transcriptional control of metabolic genes is a crucial mechanism to coordinate ROS homeostasis. Therefore, a better understanding of how ROS metabolism is regulated by specific transcription factors can contribute to uncovering new therapeutic strategies. A large body of work has positioned the estrogen-related receptors (ERRs), transcription factors belonging to the nuclear receptor superfamily, as not only master regulators of cellular energy metabolism but, most recently, of ROS metabolism. Herein, we will review the role played by the ERRs as transcriptional regulators of ROS generation and antioxidant mechanisms and also as ROS sensors. We will assess how the control of ROS homeostasis by the ERRs can be linked to physiology and disease and the possible contribution of manipulating ERR activity in redox medicine.

## 1. Introduction

Reactive oxygen species (ROS) is a generic term that includes several derivatives of molecular oxygen such as the superoxide anion radical (O_2_^•−^) and hydrogen peroxide (H_2_O_2_), two key redox signaling agents. In mammalian cells, mitochondria are an important source of ROS. The generation of ROS takes place mainly in the electron transport chain (ETC) on the inner mitochondrial membrane during oxidative phosphorylation (OXPHOS). To protect against ROS accumulation, various redox systems are operational, such as the glutathione or thioredoxin redox couples that participate in cell signaling. When ROS exceed the cellular antioxidant defense system, either through an increase in ROS levels or a decrease in the antioxidant capacity, oxidative stress occurs, a process referred to as oxidative distress. ROS engender oxidative modification of macromolecules such as nucleic acids, proteins, and lipids. In particular, oxidative stress-induced impairments in protein function may ultimately lead to cell death and has thus been implicated in a myriad of pathologies [1]. Consequently, knowledge of how cellular ROS signaling is regulated is key to understand the etiology of many diseases and explore novel possible applications of redox medicine. On the other hand, ROS also contribute to the proper functioning of the cell. The physiological role of H_2_O_2_ and associated redox-dependent signaling are referred to as oxidative eustress [2]. Indeed, H_2_O_2_ occurs at a low level in normally respiring cells and contributes to mild mitochondrial stress that leads to an enhancement of health and longevity [3]. Low levels of H_2_O_2_ also induce the reversible oxidation of proteins and therefore influences many cellular and inter-organ processes such as cell proliferation, differentiation and migration, often through direct regulation of gene expression [4]. Several transcription factors have been shown to be regulated by ROS but also to regulate the response to oxidative stress. These include nuclear factor kappa-light chain-enhancer of activator B cells (NF-κB), hypoxia inducible factor-1α (HIF-1α), nuclear factor (erythroid derived 2)-like 2 (Nrf2) and activator protein-1 (AP-1) [5,6,7,8]. Recently, the estrogen-related receptors (ERRs), a sub-class of nuclear receptors well-known as central transcriptional regulators of energy homeostasis and mitochondrial biogenesis and function [9,10], were also shown to play direct roles in the control of ROS homeostasis [11,12]. Herein, we will first highlight the properties and function of the ERRs linking them to ROS production and metabolism, then the phenomena linking ERR and ROS during oxidative eustress and distress, and lastly, we will discuss how manipulating the activity of the ERRs could potentially participate in redox medicine.

## 2. The ERRs

The ERR subfamily is part of the superfamily of nuclear receptors and comprised of three members referred to as ERRα (NR3B1), ERRβ (NR3B2), and ERRγ (NR3B3) [13,14,15,16]. Most nuclear receptors are ligand-regulated transcription factors that recognize small lipophilic hormones, vitamins and metabolites, and thus provide a direct link between extracellular signals and regulation of gene expression. However, the ERRs are referred to as orphan nuclear receptors as specific endogenous ligands are yet to be identified for these receptors. The inclusion of estrogen in their name relates only to the similarity in amino acid sequences that ERRs share with the classical estrogen receptors (ERs) [13,17]. In spite of being structurally related to the ERs, the ERRs do not bind naturel estrogens. Moreover, the ERRs and ERs recognize different binding sites on chromatin [18]. The consensus sequence of DNA recognized by the ERRs and generally present in the promoter of ERR-regulated genes has the consensus sequence TNAAGGTCA and is referred to as an estrogen-related response element (ERRE) [19,20]. While being classified as orphan receptors, the ERRs do possess a functional ligand-binding pocket proper to nuclear receptors as synthetic pharmacological agents can activate or inhibit the transcriptional activity of ERRs, indicating that the activity of the three ERR isoforms could indeed be controlled by one or more natural ligands [21,22,23,24,25]. Moreover, the three ERR members share a strong structural homology including a non-conserved amino terminal domain (NTD) that is the target of post-translational modifications and a central DNA-binding domain (DBD) composed of two zinc finger motifs that recognize ERRE sequences [9]. Several ligand-independent mechanisms have also been uncovered that regulate the activities of the ERRs. These mechanisms include direct interactions with coactivator and corepressor proteins, post-translational modifications (PTMs) and regulation of their expression by microRNAs [26,27,28,29,30,31,32,33,34,35,36,37,38,39,40,41,42]. In particular, the coactivators known as peroxisome proliferator-activated receptor γ (PPARγ)-coactivator 1 α and β (PGC-1α, β) are potent activators of all three ERR isoforms and have often been referred to as protein ligands for these receptors [43,44,45,46,47]. PGC-1α/β can interact with the ERRs via their activation function-2 (AF-2) domain which is located within the carboxy-terminal ligand-binding domain [9]. Indeed, the PGC-1/ERR transcriptional axis has been implicated in the control of vast metabolic gene networks associated with mitochondrial biogenesis, OXPHOS, fatty acid metabolism, glucose uptake, and gluconeogenesis in various tissues [48,49,50,51,52,53,54,55].

One or more members of the ERR family are expressed in every tissue, suggesting a vital role for these factors in the proper maintenance of cellular function [56]. ERRα is ubiquitously expressed and the most abundantly expressed isoform whereas ERRβ and ERRγ display more specific patterns of expression and are generally expressed at lower levels compared to ERRα. When present in the same cell, the three ERR isoforms can form heterodimers [19,57]. In support of this observation, comparison of chromatin immunoprecipitation coupled with DNA microarrays (ChIP-chip) or massively parallel sequencing (ChIP-seq) datasets revealed over 80% overlap in ERRα and ERRγ binding peaks in distinct cell types [12,19,58]. Indeed, ERR isoforms can compensate for each other’s activity as knockdown of two or more ERR isoforms are required to suppress mitochondrial biogenesis, abrogate the transcriptional response to adrenergic stimulation in brown fat and disrupt normal cardiac bioenergetics [59,60,61]. ERRα knockout mice, while displaying a variety of metabolic deficiencies, are alive and can reproduce normally [62]. Nonetheless, gene knockouts in mice have demonstrated that loss of each ERR isoform results in distinct developmental and tissue-specific phenotypes, demonstrating that each ERR isoform has also specific roles to play in the control of cellular metabolism and other biological processes [58,63,64,65]. Indeed, ERRα-null mice have been well-studied over time for their multiple metabolism-related phenotypes [58,62]. The ERRβ-null mouse model is embryonic lethal due to a placental formation defect [63]. ERRγ-null mice die shortly after birth due to a combination of heart, gastric, and renal potassium homeostatsis defects [60,64,66].

## 3. Transcriptional Control of ROS Metabolism by the ERRs

### ROS-Generating ERR Targets

ROS generation can come from a variety of intracellular compartments including mitochondria, the endoplasmic reticulum, peroxisomes, nuclei, the cytosol, plasma membranes and even extracellular spaces. ROS are mainly generated during mitochondrial OXPHOS or they can come from interactions with exogenous sources such as bacterial invasion. The initial ROS formed by the respiratory chain and other enzymatic components within mitochondria is superoxide O_2_^−^ and can be released towards both the mitochondrial matrix and the intermembrane space. The respiratory chain, which is an ETC, is composed of transmembrane protein complexes (I to IV) located in the inner mitochondrial membrane and the freely mobile electron transfer carriers ubiquinone and cytochrome c [67]. The respiratory chain allows for OXPHOS, a process involving the phosphorylation of ADP into ATP via the energy released by the oxidation of electron donors through the respiratory chain. The electrons eliminated from the Krebs cycle (tricarboxylic acid cycle, TCA) and transported by nicotinamide adenine dinucleotide hydrogen (NADH) and flavin adenine dinucleotide hydroquinone (FADH_2_) are used to feed the pumping of protons from the matrix to the intermembrane space, generating a gradient of protons through the internal mitochondrial membrane. Complexes I and III are generally regarded as the main ROS sources. Complex I can generate superoxide in the presence of NADH [68]. Complex II can also produce ROS but its importance was underestimated for a long time [69]. Complex III is considered as the main ROS producer under both basal and stress conditions [70]. Interestingly, it has been demonstrated that ERRα regulates the transcription of all of the enzymes that constitute the TCA cycle and a significant number of target genes that code for proteins involved in OXPHOS [71]. Integration of ChIP-based experiments in combination with specific gene-targeted studies revealed that ERRα binds to over 85% of the nuclear genes that code for complex I (GO:0045271), to 100% of the nuclear genes that code for complexes II (GO:0045273) and III (GO:0045275), to 75% of the nuclear genes that code for complex IV (GO:0045277) and to 66% of the nuclear genes that code for complex V (GO:0005753) [10,19,34,47,72,73,74,75]. ERRα also regulates genes that code for coenzyme Q and cytochrome c. Of note, it was reported that ERRγ targets the same set of promoters of genes encoding TCA cycle proteins and OXPHOS machinery [19]. Moreover, it has been shown that ERRα and ERRγ act collectively and in a redundant manner to promote mitochondrial oxidative capacity in brown adipose tissue [61]. Increasing ERRα expression by PGC1α overexpression leads to a rise in mitochondrial activity, found to stimulate the respiratory capacity fibroblasts [76]. Moreover, in a transgenic muscle-specific ERRγ overexpression mouse model, genes associated with oxidative metabolism are increased [77]. Furthermore, activation of the PGC-1β/ERRα axis by c-MYC in breast cancer cells induces the expression of all the enzymes involved in the TCA cycle [73]. Conversely, when ERRα activity is impaired via the use of knockout mice [47,78,79] or knockdown in cells [71,80], mitochondrial integrity, activity and ATP production have been demonstrated in different tissues to be decreased. In addition, genetic and pharmacological silencing of ERRα activity in mouse liver downregulates the expression of TCA cycle genes leading to the accumulation of various TCA intermediates and hepatic hyperlipidemia [34]. Interestingly, this function of the ERRs is profoundly conserved as a mutant of the drosophila ortholog of ERR (dERR) exhibits a similar reduction in TCA intermediates as well as a decrease in ETC components, resulting in a two-fold decrease in ATP levels in second-instar larvae [81,82].

**Figure 1:**
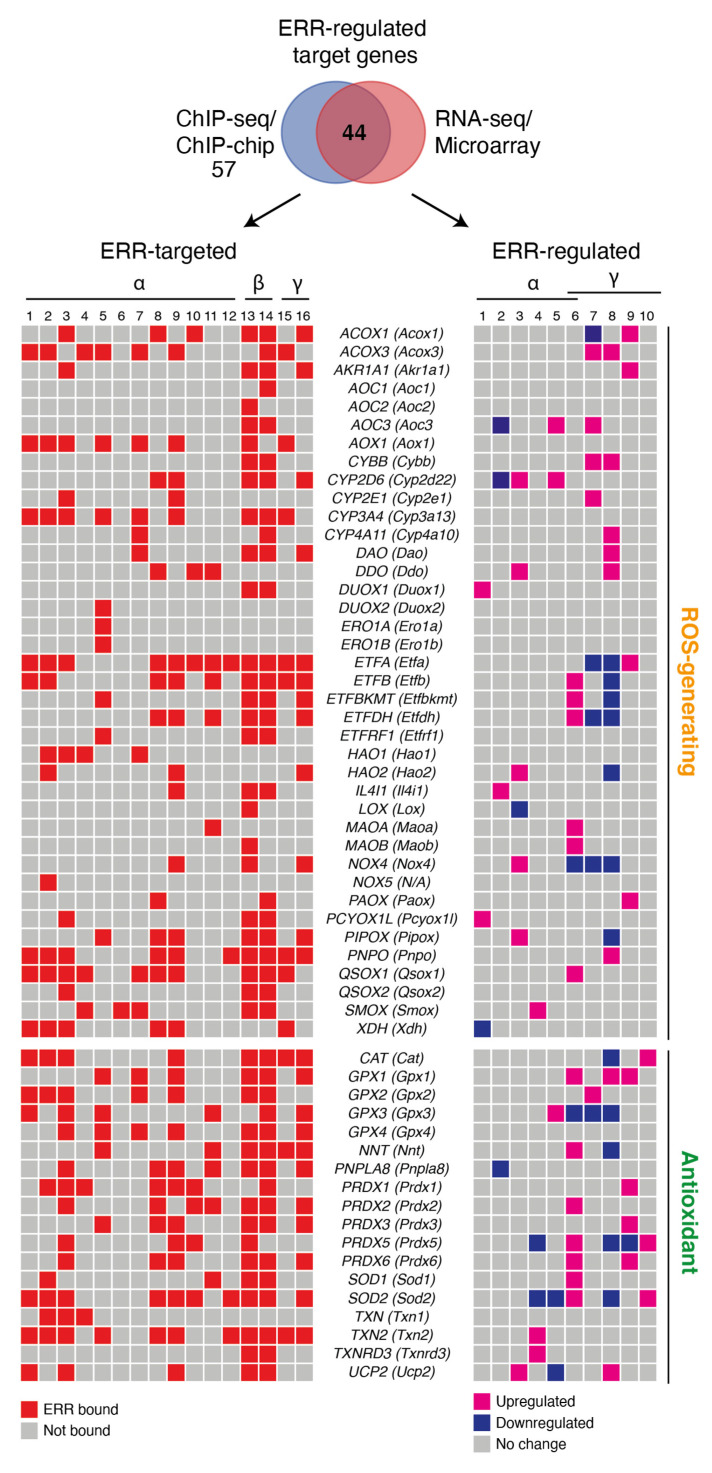
ERR-targeted enzymes implicated in ROS metabolism. Schemes follow the same formatting. Both human and mouse gene names are shown, the latter in parentheses. Genes implicated in both ROS generation and antioxidant response were manually curated from the literature and only genes found targeted (58 out of 63) by the estrogen-related-receptors (ERRs) in at least one cell or tissue from ChIP-based technologies are shown in a heatmap representation. Of the 58 ERR-targeted genes, those found differentially regulated by the ERRs based on available RNA-seq and microarray datasets are shown in a heatmap representation. Red boxes indicate ERR-occupied genes. Pink and blue boxes indicate upregulated and downregulated genes, respectively, that are ERR-regulated. For ChIP-seq, genes with binding peaks within ±20 kb from the transcriptional start sites were considered. For ChIP-chip, genes with a cutoff of *p* < 0.001 were considered. The ERR-targeted datasets shown are ordered as follows: (1) BCa-BT474 ERRα ChIP-seq (untreated) [48]; (2) BCa-BT474 + AICAR ERRα ChIP-seq (AICAR-treated) [48]; (3) BCa-SKBR3 ERRα ChIP-seq (untreated, serum-starved) [11]; (4) BCa-MCF7 ERRα ChIP-seq (untreated, serum-starved) [Deblois G, unpublished]; (5) B lymphocytes-GM12878 ERRα ChIP-seq [88]; (6) CRC-LoVo ERRα ChIP-seq [89]; (7) HCC-HepG2 ERRα ChIP-seq [90]; (8) mouse liver ERRα ChIP-chip [71]; (9) mouse liver ERRα ChIP-seq [34]; (10) mouse kidney ERRα ChIP-chip [91]; (11) mouse skeletal muscle ERRα ChIP-chip (gastrocnemius and soleus) [Dufour CR, unpublished]; (12) mouse frontal cortex ERRα ChIP-seq [92]; (13) mouse ESC ERRβ ChIP-seq [93]; (14) mouse ESC ERRβ ChIP-seq [94]; (15) BCa-BT474 ERRγ ChIP-seq [12]; (16) mouse kidney ERRγ ChIP-seq [95]. The ERR-regulated datasets shown are ordered as follows: (1) BCa-SKBR3 siERRa vs. siCtrl microarray [11] cutoff: *p* < 0.05; (2) BCa-SKBR3 siERRa vs. siCtrl microarray (untreated, serum-starved) cutoff: *p* < 0.05 [11]; (3) mouse kidney ERRα KO vs. WT (basal) microarray [91] cutoff: FC 1.2 and *p* < 0.05; (4) mouse skeletal muscle ERRα KO vs. WT (basal) microarray (gastrocnemius) [96] cutoff: FC 1.2 and *p* < 0.05; (5) mouse heart ERRα KO vs. WT (basal) microarray [19] *p* < 0.05; (6) mouse cardiac ventricles ERRα/γ DKO vs. WT (basal) microarray [97] cutoff: FC 1.5 and *p* < 0.05; (7 and 8) mouse RECs ERRα KO vs. WT (basal) RNA-seq in 3-month (7) or 3-week (8) old mice [95] cutoff: logFC1.5 and *p* adjusted < 0.05; (9 and 10) PCa-LNCaP siERRγ vs. siCtrl basal (9) or R1881 (10) (synthetic androgen) microarray [98] *p* < 0.05. Abbreviations used: AICAR, 5-aminoimidazole-4-carboxamide ribonucleotide); BCa, breast cancer; CRC, colorectal cancer; Ctrl, control; DKO, double knockout; ESC, embryonic stem cell; FC, fold-change; HCC, hepatocellular carcinoma; KO, knockout; N/A, non-applicable; PCa, prostate cancer; REC, renal epithelial cells; WT, wild type.

Another endogenous source of superoxide is owned to nicotinamide adenine dinucleotide phosphate (NADPH) oxidases (NOX), enzymes that catalyze the production of O_2_^−^ or H_2_O_2_ using NADPH as a reductant. For many years, superoxide generation through NOX was thought to occur only in phagocytes. However, the seven members of the NOX family have various tissue distributions and activation mechanisms [83]. Cross-examination of genome-wide location analyses found Nox4 and Nox5 as ERR target genes (Figure 1).

**Figure 2:**
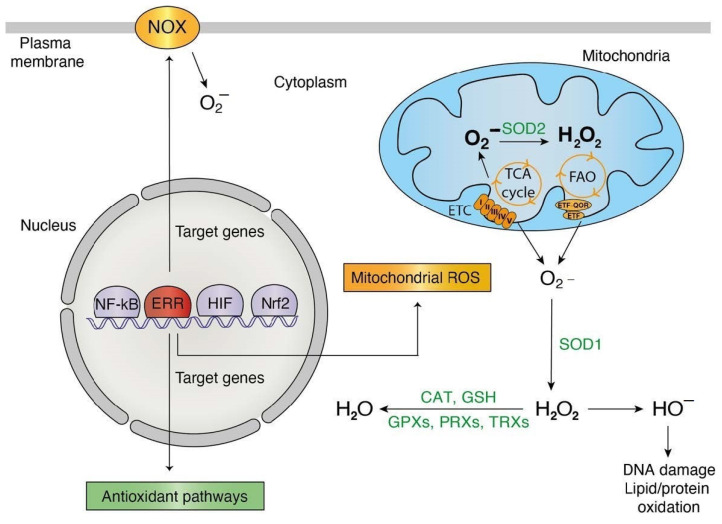
ERR pleiotropic roles in redox signaling. Orange: phenomena/actors implicated in ROS generation and transcriptionally targeted by the estrogen-related-receptors (ERRs). Green: phenomena/actors implicated in ROS detoxification and transcriptionally targeted by the ERRs. Abbreviations used: CAT, catalase; ETC, electron transport chain; FAO, fatty acid oxidation; GPX, glutathione peroxidase; GSH, glutathione; NOX, NADPH oxidase; PRX, peroxiredoxin; SOD, superoxide dismutase; TCA cycle, tricarboxylic acid cycle; TRX, thioredoxin.

The passage of electrons from fatty acids to the ETC is also a source of reducing equivalents responsible for increasing the ROS production chain due to the actions of electron transfer flavoprotein (ETF) and fatty acid β-oxidation (FAO) [84]. The first dehydrogenase reaction in FAO by acyl-CoA dehydrogenases (ACAD) generates FADH_2_, with the electrons donated to ETF. ETF is linked to ETF-coenzyme Q oxidoreductase (ETF-QOR) and provides an alternative pathway to pass electrons to coenzyme Q in the ETC [84]. During these reactions, some superoxide can be produced. Remarkably, the first identified genomic target of ERRα was Acyl-CoA dehydrogenase medium chain (*ACADM*), which codes for the enzyme that catalyzes the first stage of FAO [20,85]. Further genomic profiling experiments not only confirmed the recruitment of ERRα to the ACADM promoter in vivo but also to over 65% of genes implicated in FAO (GO:0006635) [19,34]. In response to ERRα overexpression, additional genes encoding enzymes of mitochondrial very long chain acyl-coenzyme A dehydrogenase (*ACADVL*) and long-chain hydroxyacyl coenzyme A dehydrogenase (*HADHA*) are also activated in parallel with ACADM [79]. Conversely, in brown adipocytes, FAO was decreased by 30–50% in ERRα knockout mice [86]. Additional ERRα targets include genes that code for the two subunits of ETF (*ETFA* and *ETFB*), the gene encoding ETF-QOR (*ETFDH*), and to one regulator of the electron transfer flavoprotein (*ETFBKMT*) (Figure 1). In an interesting way, these genes have been shown to be upregulated in cardiac ventricles of ERRα knockout mice and downregulated in renal epithelial cells of ERRγ knockout mice (Figure 1). The ERRs can also target genes encoding a variety of enzymes that are generating ROS. These include acyl-CoA oxidase (*ACOX1*), involved in peroxisome β-oxidation, D-aspartate oxidase (*DDO*) that participates in alanine and aspartate metabolism, hydroxyacid oxidase (*HAO1*) that oxidizes glycolic acid to glyoxylate and glyoxylate into oxalate, pyridoxamine 5′-phosphate oxidase (*PNPO*) that catalyzes several reactions in the vitamin B6 metabolic pathway, and xanthine dehydrogenase (*XDH*) involved in the oxidative metabolism of purines (Figure 1). Most of these genes have been shown to be differentially regulated by ERRs in at least one tissue or cell line in the current literature (Figure 1). In summary, the ERRs possess the potential to regulate at the transcriptional level all the mitochondrial actors of ROS production including the TCA cycle, ETC complexes I, II and III, β-oxidation and the ETF complexes as well as a large number of ROS-generating enzymes (Figure 1 and Figure 2). These findings clearly indicate that the ERRs play a pleiotropic role as transcription factors in the regulation of ROS metabolism3.2. ROS-Counteracting ERR Targets.

There is a variety of enzymatic and nonenzymatic scavengers that serve to counterbalance the effects of oxidants. For enzymatic antioxidants, superoxide dismutase (SODs), catalase (CAT), glutathione peroxidase (GPx) and redox proteins such as thioredoxins (TRXs) and peroxiredoxins (PRXs), have been found to play crucial roles in the antioxidant defenses [87]. The enzymatic antioxidants can directly or indirectly catabolize ROS to protect the cells. Once again, the ERRs have been found to bind to regulatory regions of genes encoding the vast majority of the components of antioxidant defense systems (Figure 1 and Figure 2). A wide variety of these enzymes have been shown to be upregulated or downregulated in several contexts including ERRα-null mouse heart, kidney, and skeletal muscle, mouse ERRγ-null kidney, ERRα-knockdown in breast cancer cells and ERRγ-knockdown in prostate cancer cells (Figure 1). The superoxide dismutase family is composed of three members, CuZn-SOD (superoxide dismutase Cu–Zn), Mn-SOD (superoxide dismutase Mn, mitochondrial) and EC-SOD (extracellular superoxide dismutase Cu-Zn). Mn-SOD is localized in the mitochondrial matrix whereas EC-SOD is primarily localized in the extracellular matrix. They allow for the detoxification of superoxide O_2_− by their conversion into H_2_O_2_. The three ERRs directly target and regulate SOD2 that codes for MnSOD [11] and SOD1 in different tissues and cell lines (Figure 1). Moreover, ERRα regulates SIRT3 which in turn increases the expression of the ROS-detoxifying enzyme SOD2 to decrease ROS levels [87].

H_2_O_2_ produced by the action of SODs is reduced to water by CAT and GPx. The genes encoding these enzymes are targeted and regulated by the ERRs (Figure 1). Members of the GPx family have anti-oxidative functions within different cellular components. GPx oxidizes reduced glutathione (GSH) to glutathione disulfide (GSSG) during oxidative stress [99]. GSH is also a key determinant of redox signaling that regulates cell proliferation, apoptosis and immune function. Recently, our group showed that pharmacological inhibition of ERRα leads to an induction of glutamine-driven glutathione production and suppression of ROS in breast cancer cells via upregulation of ERRγ activity [12]. The presence of ERRγ on DNA may be necessary for ERRα recruitment to certain genes since the ERRγ inverse agonist GSK5182 also suppresses ERRα binding to target genes [12]. Therefore, ERRα and ERRγ, as ROS sensors, are codependent and rely on each other’s activity to induce an antioxidant response. The exact mechanisms by which the ERRs act as cellular ROS sensors remain to be defined. Deblois et al. also showed that pharmacological inhibition of ERRα activity in a breast cancer cell line resistant to epidermal growth factor receptor inhibition by the anti-cancer drug lapatinib leads to reduced detoxification capacity and increased oxidative damage [11]. The role of the ERRs in ROS detoxification by glutathione is therefore context- and tissue-dependent. Disposal of H_2_O_2_ is also closely associated to the TRX and PRX antioxidant systems. The TRX system is composed of NADPH, thioredoxin reductase (TrxR) and thioredoxin (Trx) [100]. Trx exists in two isoforms, cytosolic Trx1 (Txn1) and mitochondrial Trx2 (Txn2), whose genes are targeted by the ERRs and Txn2 has been shown to be upregulated by loss of ERRα in mouse skeletal muscle (Figure 1). The PRX system is composed of six members, Prx1–6 (Prdx1–6). Prdx1, 2, and 4 are found primarily in the cytoplasm but are also expressed in nuclei. Prdx3 and Prdx5 are localized in the mitochondria, but Prdx5 has also been found in the cytoplasm, nuclei and peroxisomes [101]. Prdx1–6, with the exception of Prdx4, were all shown to be ERR targets in several tissues and cell lines and found differentially regulated by manipulation of ERR activity (Figure 1). PRXs can transmit the oxidizing equivalents from H_2_O_2_ to other target proteins. Both the GSH and TRX/PRX system can protect against oxidative stress via the efficient removal of various ROS.

Energetic substrates involved in several specific metabolic pathways can generate redox cofactors (NADH and FADH) that can be readily used to maintain or restore redox homeostasis. These metabolic pathways include glycolysis, glutaminolysis, FAO, one-carbon metabolism and the pentose phosphate pathway [102], all found targeted by the ERRs in breast cancer cells in a recent study [12].

Other antioxidant systems, such as mitochondrial nicotinamide nucleotide transhydrogenase (NNT), can also reduce H_2_O_2_ in water by shifting reducing equivalents from NADH to NADPH, thereby supporting and strengthening the capacity of the TRX and GSH systems [103]. The NNT-encoding gene is also an ERR target and has been shown to be upregulated in cardiac ventricles of ERRα/γ double knockout mice and downregulated in the renal epithelium cells of ERRγ-null mice (Figure 1). Furthermore, the mitochondrial Ca^2+^ independent phospholipase A2γ (iPLA2γ) plays an antioxidant role in various tissues [104,105,106]. During oxidative stress, H_2_O_2_-activated phospholipase iPLA2γ together with UCP2 provides an antioxidant function by releasing free fatty acids upon the cleavage of lipids. UCP2 then attenuates mitochondrial superoxide formation by mild uncoupling [105]. Uncoupling diminishes the production of mitochondrial O_2_^−^. The iPLA2γ-encoding genes *PNPLA8* and *UCP2* are both ERR-targeted genes (Figure 1).

Further evidence supporting that the ERRs not only target these genes but also modulate their regulation is that PGC-1α, whose action on gene expression depends essentially on the ERRs, was shown to increase the levels of MnSOD/SOD2, CAT, PRX3/5, UCP2, TRXR2, and TRX2 [107,108,109]. In fact, PGC-1α upregulation promotes cell survival by protecting cancer cells from excessive mitochondrial ROS generation [110,111]. It is interesting to note that the antioxidant function of the ERRs is associated with their roles enhancing mitochondrial electron transport in cells with high energy demands. Therefore, the potent regulation of antioxidant defense through the ERRs is likely to ensure an adequate response to the cytotoxic effects of ROS accumulation promoted via the active ERR-dependent pathways involved in cellular energy production.

## 4. Influence of ROS in ERR Activity

One property of members of the nuclear receptor family is to be part of feedback or feedforward loops regulating their activity. The previous section clearly established that a major function of the ERRs is to regulate a vast array of genes that affect the concentration of ROS within cells. Consequently, it could be assumed that ROS themselves could in turn affect the activity of the ERRs. In most cases, ROS signaling can regulate transcription factors by inducing reversible and irreversible PTMs like cysteine modifications or by direct interactions with redoxins. These proteins are involved in redox reactions and can reverse some oxidative stress-induced PTMs on cysteine thiols [112]. Several redox sensors have been identified to transduce the signal to their respective transcription factors [113]. During oxidative stress, it was shown that a change occurs in the levels of expression of two ERR isoforms [12]. While the activity and levels of these two ERR isoforms are clearly redox-sensitive, the exact mechanisms by which this mode of regulation occurs are currently unknown. However, nuclear receptors contain redox-active thiol groups and changes in cellular redox signaling can affect the ability of these nuclear receptors to regulate gene expression. In particular, it has been shown that thiol oxidation negatively regulates ligand and DNA binding for a number of nuclear receptors, including the closely related ERs [114]. Since nuclear receptors share a conserved molecular architecture, it can be speculated that redox-dependent mechanisms that regulate the activity of the ERRs could be a feature common to at least a subset of nuclear receptors involved in the regulation of cellular energy metabolism.

## 5. ERR Activities and ROS during Oxidative Eustress and Distress

Intracellular ROS concentration is a fine equilibrium that enables redox signaling under eustress (physiological) or distress (pathological) conditions that causes mitochondrial damage and cell death. In the next section, we will explore the activities of the ERRs in these two contexts.

### 5.1. ERRs and Oxidative Eustress

Low levels of ROS production, and therefore mild mitochondrial stress, has been shown to play an important role in protecting health span and longevity by induction of a process referred to as mitohormesis. Indeed, mitochondria can initiate and transduce signals to the nucleus. These signals coordinate a transcriptional response resulting in both mitochondrial and non-mitochondrial adaptations necessary to maintain cellular homeostasis. For example, mild increased mitochondrial ROS caused by ETC mutations or knockdown of SOD2 extends lifespan in the worm *Caenorhabditis elegans* and mice [115,116]. Interestingly, mild mitochondrial stress in liver of a mouse model of inducible *Sod2* knockdown results in the activation of mitochondrial signaling pathways which are dependent on the key ERR co-activator, PGC-1α [116]. Furthermore, activation of mitochondrial biogenesis by PGC-1α through the ERRs is downstream of the oxidant level-regulated AMP kinase (AMPK) [117]. AMPK induces mitochondrial fusion, which may be important as this process prevents mitochondrial dysfunction and excessive ROS formation [118]. These observations demonstrate the diversity in mechanisms to directly connect ROS levels and transcriptional regulation of mitochondrial networks by the ERRs. The mitochondrional network is characterized by a large polymorphism of shapes as mitochondria form a dynamic complex maintained in balance through a regulated equilibrium between fusion and fission. The dynamin family of GTPases regulates mitochondrial dynamics. Interestingly, during oxidative eustress, mitochondrial dynamics is tightly coupled to ROS signaling [119,120]. Aberrant mitochondrial morphology can lead to enhanced ROS formation, which in turn may deteriorate mitochondrial health and further exacerbate oxidative stress in a feedforward cycle. As expected, a large number of genes that code for actors implicated in mitochondrial dynamics are targets of ERR. First, fusion of the outer and inner mitochondrial membrane is mediated by mitofusin-1 and mitofusin-2 (MFN1 and MFN2) and optic atrophy 1 (OPA1) [121,122,123], and their genes are all ERRα targets [34,124,125]. Second, it is interesting to note that during physical exercise when the muscle requires sufficient energy, expression of *MFN2* is increased in skeletal muscle in an ERRα-dependent manner [124]. Third, a study showed that *MFN2* expression is also upregulated in skeletal muscle and brown adipose tissue by cold exposure or by treatment with the β3-adrenergic agonist, and this upregulation is dependent on direct ERRα binding to the *MFN2* promoter [125]. Fourth, ERRα chemical inhibition increases mitochondrial fragmentation in cultured renal tubular cells, possibly via the predominant mitochondrial fission process with decreased mitofusin-2 expression [126]. Notably, the fission machinery of the outer membrane is composed of dynamin-related protein 1 (DRP1) and dynamin-2 (DYN2) [127,128], and their genes (*Dnm1l* and *Dnm2*) are components of the ERRα-regulated mitochondrial biogenesis gene network. ERRα has also been shown to induce the expression of the autophagy-regulating kinase ULK1, thus promoting DRP1-mediated mitochondrial fission [80].

### 5.2. ERRs in Oxidative Distress

One of the most vivid consequences of oxidative distress is apoptosis due to the opening of the mitochondrial permeability transition pore. Several essential players of apoptosis, including pro-caspases, cytochrome *c* or apoptosis-inducing factor (AIF), are released into the cytosol. In this context, ERRα-null hepatocytes have been shown to have a defective apoptotic program that favors necrosis and inflammation [78]. In contrast, ERRα overexpression has been shown to increase apoptosis in mesangial cells of the kidney [129]. In this study, the authors showed that ERRα overexpression induces a decrease in the expression of the antiapoptotic gene *BCL-2*, whereas the proapoptotic markers BAX and caspase-3 were significantly increased. At the same time, in mesenchymal stem cells, the PGC-1α/ERRα axis upregulates the expression of Bcl-2 and promotes cell survival [130]. Furthermore, in endometrial cancer cell lines, upregulation of ERRα has been recently shown to inhibit apoptosis through Bcl-2 upregulation and caspase-3 downregulation [131]. Thus, the anti- or pro-apoptotic function of the ERRs is likely context- and tissue-dependent and could be related to ROS signaling. Indeed, ChIP-seq [34] and gene expression [131] profiling have shown that ERRα binds to and regulates the expression of numerous genes implicated in apoptosis including antiapoptotic (e.g., *BCL2, MCL1, BCL2L1, BCL2L2*) and proapoptotic (e.g., *BMF*, *BIK*, *BNIP3*, *BCL2L14*, *BBC3*, *BCL2L13*) Blc2 family members. ERRα also targets the caspase-3 gene (*CASP3*) as well as *VDAC1-3,* which encode VDAC proteins, which are members of the mitochondrial permeability transition pore (mPTP), whilst their roles in this process remain controversial.

A hypoxic state occurs when cells have low oxygen levels below that of normal levels referred to as normoxia. Hypoxia and associated oxidative distress both lead to a reprogramming of energy metabolism transcriptional programs. While it is still a matter of debate, it has been hypothesized that hypoxia could promote ROS levels by acting on complexes I, II, and III of the ETC. One major cellular adaptive response to hypoxia involves the stabilization and activation of hypoxia-inducible factor (HIF), a key factor controlling the transcriptional networks involved in reprogramming energy metabolism. HIF-1 is a heterodimeric transcription factor consisting of a constitutively expressed β-subunit (HIFβ) and an oxygen-regulated α-subunit (HIFα). HIFα has two isoforms, HIF-1α and HIF-2α that have distinct roles despite sharing an important homology in structure [132]. Ao et al. previously reported direct interactions between ERR family members and HIF-1 requiring the AF-2 domain of ERRα [133]. This study showed that the three ERR family members can interact with the functional HIF-1 heterodimer, but not with the individual HIF-1α or HIF-1β subunit. ERRα–HIF-1 interaction could inhibit ubiquitination of HIF-1α and thus reduce its degradation. More recently, Zou et al. showed that ERRα could physically interact with HIF-1α in a prostate cancer cell line [134]. An interaction that is conserved in drosophila since dERR was also shown to interact with dHIFa [135]. Moreover, Zou et al. reported that ERRα overexpression enhances HIF-1α protein expression by suppressing HIF-1α ubiquitination [134]. This result suggests that ERRα could function to pre-adapt cancer cells to meet hypoxia-induced stress. In bone-marrow derived macrophages, loss of ERRα promoted HIF-1α stabilization [136]. Interestingly, in neural cell-derived tumors, the transcription of *HIF2A* is regulated by ERRα under normoxia and hypoxia. Indeed, inhibition of ERRα decreases *HIF2A* mRNA expression and high expression of the ERRα-encoding gene *ESRRA* significantly correlates with poor overall and progression-free survival in neuroblastoma [137]. In skeletal muscle, HIF-2α is also regulated by the PGC-1α/ERRα axis during the response to exercise and during fiber-type switching [138]. In brief, the ERRs may play a key transcriptional role in response to hypoxia via their interactions with HIF family members.

## 6. ERRs in Oxidative Stress-Related Physiopathology

Dysregulation of ROS production has been implicated in the development of various chronic disorders such as cancer as well as respiratory, neurodegenerative and digestive diseases. The role that ROS play in disease settings is well studied and can take different forms by acting either positively or negatively in each distinct disease [1].

**Figure 3:**
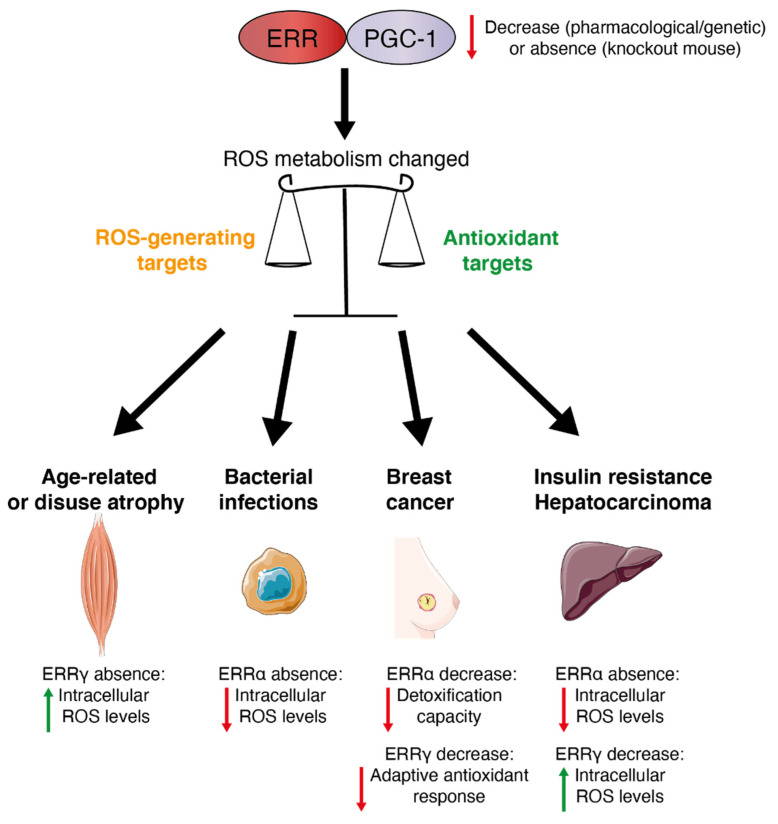
The PGC-1/ERR axis: a redox medicine strategy. The PGC-1/ERR axis plays a role in the balance of ROS metabolism and can be an attractive target to manage several oxidative stress-related physiopathologies. Red arrows indicate a decrease, green arrows indicate an increase.

In cancer, ROS have a role in apoptosis of tumor cells as ROS production increases with higher demand for cellular energy for growth and proliferation. Moreover, chemotherapeutic agents provoke cytotoxic effects on cancer cells via the induction of oxidative stress and associated cellular damage. ROS can also incite metastasis and radioresistance via mechanisms to re-establish redox balance fueling drug resistance and cell survival. For example, the ERRγ inverse agonist GSK5182 has been shown to inhibit an adaptive antioxidant ERR-driven response thereby accentuating the chemotherapy-induced cytotoxic effects of paclitaxel in breast cancer cells [12]. Moreover, pharmacological inhibition of ERRα activity in a breast cancer cell line that is resistant to lapatinib treatment leads to reduced detoxification capacity and increased oxidative damage [11]. Therefore, pharmacological inhibition of ERRα re-sensitizes the cells to lapatinib treatment leading to cell death. Moreover, inhibition of ERRγ by GSK5182 was also found to suppress tumorigenesis of hepatocellular carcinoma in part by inducing ROS [139]. In contrast, loss of ERRα in mice exacerbated the development of hepatocellular carcinoma induced by the carcinogen diethylnitrosamine which was associated with decreased ROS generation [78]. These differences of action between ERRα inhibition in breast cancer and hepatocarcinoma could be explained by a tissue-specific effect or acute loss (pharmacological) versus the chronic loss (knockout mice) of ERRα. ERRs have been studied in other pathological contexts (Figure 3). For example, during bacterial infections, ERRα has been shown to be a key component of the innate immune response against intracellular bacterial pathogens [47,140]. Indeed, ERRα-deficient macrophages showed lower intracellular ROS levels. The PGC-1α/ERRα axis promotes mitochondrial function during macrophage activation and limits ROS production. Moreover, in myocytes of muscle-specific ERRγ^−/−^ mice, ROS production was significantly elevated due to mitochondrial defects and this ROS increase impaired myotube formation through activation of skeletal muscle atrophy pathways [141]. Therefore, the ERRγ pathway may have a protective role in age-related or disuse atrophy due in part to its role in ROS homeostasis. Oxidative stress also plays a key role in the development of diabetes-related complications. Indeed, mitochondrial dysfunction is associated with diabetes, and this mitochondrial impairment may trigger oxidative stress [142,143]. It has been shown that pharmacological inhibition of ERRα in a diet-induced model of obesity improved insulin sensitivity [23] and similar results have been obtained in an ERRα-null mouse model [144]. Therefore, it will be interesting to study ERRα regulation of ROS metabolism in the context of insulin resistance.

## 7. Conclusions and Perspectives

In this review, we have presented a large body of evidence that the ERRs play pleiotropic roles in the transcriptional control of ROS metabolism. The ERRs can have both anti- and pro-oxidant effects. Indeed, we have documented that while the ERRs target and regulate a large array of genes encoding antioxidant enzymes, they also contribute to the production of cellular ROS via transcriptional control of mitochondrial biogenesis and the ETC. Moreover, we have highlighted potential roles for the ERRs during both oxidative eustress and distress, notably attributed to mitohormesis and mitochondrial dynamics and apoptosis and hypoxia, respectively. As expected, a high degree of complexity characterizes ROS interactions with ERR-regulated pathways, as ROS affect cellular functions via numerous mechanisms and organelles. Another hurdle to our current understanding of ROS–ERR interactions is that many of these interactions and downstream effects appear to be cell type- and context-specific. Nonetheless, it has become apparent that members of the ERR family should be added to the short list of transcription factors that exert great influence on ROS metabolism and biology. It is therefore hoped that a greater understanding of the complex interplay between ERR family members and oxidative stress may lead to the development of therapeutic strategies in redox medicine for the treatment of human diseases.

## References

[1] Yang S., Lian G. (2020). ROS and Diseases: Role in Metabolism and Energy Supply. Mol. Cell. Biochem..

[2] Sies H. (2017). Hydrogen Peroxide as a Central Redox Signaling Molecule in Physiological Oxidative Stress: Oxidative Eustress. Redox Biol..

[3] Ristow M. (2014). Unraveling the Truth About Antioxidants: Mitohormesis Explains ROS-induced Health Benefits. Nat. Med..

[4] Shadel G.S., Horvath T.L. (2015). Mitochondrial ROS Signaling in Organismal Homeostasis. Cell.

[5] Lingappan K. (2018). NF-kappaB in Oxidative Stress. Curr. Opin. Toxicol..

[6] Movafagh S., Crook S., Vo K. (2015). Regulation of Hypoxia-inducible Factor-1a by Reactive Oxygen Species: New Developments in an Old Debate. J. Cell. Biochem..

[7] Kasai S., Shimizu S., Tatara Y., Mimura J., Itoh K. (2020). Regulation of Nrf2 by Mitochondrial Reactive Oxygen Species in Physiology and Pathology. Biomolecules.

[8] Turpaev K.T. (2002). Reactive Oxygen Species and Regulation of Gene Expression. Biochemistry.

[9] Giguère V. (2008). Transcriptional Control of Energy Homeostasis by the Estrogen-related Receptors. Endocr. Rev..

[10] Eichner L.J., Giguère V. (2011). Estrogen Related Receptors (ERRs): A New Dawn in Transcriptional Control of Mitochondrial Gene Networks. Mitochondrion.

[11] Deblois G., Smith H.W., Tam I.S., Gravel S.P., Caron M., Savage P., Labbé D.P., Bégin L.R., Tremblay M.L., Park M. (2016). ERRα Mediates Metabolic Adaptations Driving Lapatinib Resistance in Breast Cancer. Nat. Commun..

[12] Vernier M., Dufour C.R., McGuirk S., Scholtes C., Li X., Bourmeau G., Kuasne H., Park M., St-Pierre J., Audet-Walsh E. (2020). Estrogen-related Receptors are Targetable ROS sensors. Genes Dev..

[13] Giguère V., Yang N., Segui P., Evans R.M. (1988). Identification of a New Class of Steroid Hormone Receptors. Nature.

[14] Eudy J.D., Yao S.F., Weston M.D., Ma-Edmonds M., Talmadge C.B., Cheng J.J., Kimberling W.J., Sumegi J. (1998). Isolation of a Gene Encoding a Novel Member of the Nuclear Receptor Superfamily from the Critical Region of Usher Syndrome Type IIa at 1q41. Genomics.

[15] Hong H., Yang L., Stallcup M.R. (1999). Hormone-independent Transcriptional Activation and Coactivator Binding by Novel Orphan Nuclear Receptor ERR3. J. Biol. Chem..

[16] Heard D.J., Norby P.L., Holloway J., Vissing H. (2000). Human ERRgamma, a Third member of the Estrogen Receptor-related Receptor (ERR) Subfamily of Orphan Nuclear Receptors: Tissue-specific Isoforms are Expressed During Development and in the Adult. Mol. Endocrinol..

[17] Tremblay A.M., Giguère V. (2007). The NR3B Subgroup: An ovERRview. Nucl. Recept. Signal.

[18] Deblois G., Hall J.A., Perry M.C., Laganière J., Ghahremani M., Park M., Hallett M., Giguère V. (2009). Genome-wide Identification of Direct Target Genes Implicates Estrogen-related Receptor α as a Determinant of Breast Cancer Heterogeneity. Cancer Res..

[19] Dufour C.R., Wilson B.J., Huss J.M., Kelly D.P., Alaynick W.A., Downes M., Evans R.M., Blanchette M., Giguère V. (2007). Genome-wide Orchestration of Cardiac Functions by Orphan Nucler Receptors ERRα and γ. Cell Metab..

[20] Sladek R., Bader J.-A., Giguère V. (1997). The Orphan Nuclear Receptor Estrogen-related Receptor α is a Transcriptional Regulator of the Human Medium-chain Acyl Coenzyme A Dehydrogenase Gene. Mol. Cell. Biol..

[21] Kallen J., Lattmann R., Beerli R., Blechschmidt A., Blommers M.J., Geiser M., Ottl J., Schlaeppi J.M., Strauss A., Fournier B. (2007). Crystal Structure of Human Estrogen-related Receptor Alpha in Complex with a Synthetic Inverse Agonist Reveals Its Novel Molecular Mechanism. J. Biol. Chem..

[22] Greschik H., Flaig R., Renaud J.P., Moras D. (2004). Structural Basis for the Deactivation of the Estrogen-related Receptor Gamma by Diethylstilbestrol or 4-hydroxytamoxifen and Determinants of Selectivity. J. Biol. Chem..

[23] Patch R.J., Searle L.L., Kim A.J., De D., Zhu X., Askari H.B., O’Neill J.C., Abad M.C., Rentzeperis D., Liu J. (2011). Identification of Diaryl Ether-based Ligands for Estrogen-related Receptor α as Potential Antidiabetic Agents. J. Med. Chem..

[24] Tremblay G.B., Bergeron D., Giguère V. (2001). 4-hydroxytamoxifen is an Isoform-specific Inhibitor of Orphan Estrogen-receptor-related (ERR) Nuclear Receptors β and γ. Endocrinology.

[25] Kim J., Im C.Y., Yoo E.K., Ma M.J., Kim S.B., Hong E., Chin J., Hwang H., Lee S., Kim N.D. (2016). Identification of Selective ERRγ Inverse Agonists. Molecules.

[26] Crevet L., Vanacker J.M. (2020). Regulation of the Expression of the Estrogen Related Receptors (ERRs). Cell. Mol. Life Sci..

[27] Zhao Y., Li Y., Lou G., Zhao L., Xu Z., Zhang Y., He F. (2012). miR-137 Targets Estrogen-related Receptor α and Impairs the Proliferative and Migratory Capacity of Breast Cancer Cells. PLoS ONE.

[28] Lu T.M., Lu W., Zhao L.J. (2017). MicroRNA-137 Affects Proliferation and Migration of Placenta Trophoblast Cells in Preeclampsia by Targeting ERRalpha. Reprod. Sci..

[29] Ji H.L., Song C.C., Li Y.F., He J.J., Li Y.L., Zheng X.L., Yang G.S. (2014). miR-125a Inhibits Porcine Preadipocytes Differentiation by Targeting ERRalpha. Mol. Cell. Biochem..

[30] Lu M., Ding K., Zhang G., Yin M., Yao G., Tian H., Lian J., Liu L., Liang M., Zhu T. (2015). MicroRNA-320a Sensitizes Tamoxifen-resistant Breast Cancer Cells to Tamoxifen by Targeting ARPP-19 and ERRgamma. Sci. Rep..

[31] Liu R.H., Meng Q., Shi Y.P., Xu H.S. (2018). Regulatory Role of MicroRNA-320a in the Proliferation, Migration, Invasion, and Apoptosis of Trophoblasts and Endothelial Cells by Targeting Estrogen-related Receptor Gamma. J. Cell. Physiol..

[32] Cheng X., Du J., Shen L., Tan Z., Jiang D., Jiang A., Li Q., Tang G., Jiang Y., Wang J. (2018). miR-204-5p Regulates C2C12 Myoblast Differentiation by Targeting MEF2C and ERR. Biomed. Pharmacother..

[33] Ren Y., Jiang H., Ma D., Nakaso K., Feng J. (2011). Parkin Degrades Estrogen-related Receptors to Limit the Expression of Monoamine Oxidases. Hum. Mol. Genet..

[34] Chaveroux C., Eichner L.J., Dufour C.R., Shatnawi A., Khoutorsky A., Bourque G., Sonenberg N., Giguère V. (2013). Molecular and Genetic Crosstalks Between mTOR and ERRα are Key Determinants of Rapamycin-induced Non-alcoholic Fatty Liver. Cell Metab..

[35] Yang Y., Li S., Li B., Li Y., Xia K., Aman S., Yang Y., Ahmad B., Zhao B., Wu H. (2021). FBXL10 Promotes ERRalpha Protein Stability and Proliferation of Breast Cancer Cells by Enhancing the Mono-ubiquitylation of ERRalpha. Cancer Lett..

[36] Tremblay A.M., Wilson B.J., Yang X.J., Giguère V. (2008). Phosphorylation-dependent Sumoylation Regulates ERRα and γ Transcriptional Activity Through a Synergy Control Motif. Mol. Endocrinol..

[37] Vu E.H., Kraus R.J., Mertz J.E. (2007). Phosphorylation-dependent Sumoylation of Estrogen-related Receptor α1. Biochemistry.

[38] Wilson B.J., Tremblay A.M., Deblois G., Sylvain-Drolet G., Giguère V. (2010). An Acetylation Switch Modulates the Transcriptional Activity of Estrogen-related Recetpor α. Mol. Endocrinol..

[39] Misra J., Kim D.K., Jung Y.S., Kim H.B., Kim Y.H., Yoo E.K., Kim B.G., Kim S., Lee I.K., Harris R.A. (2016). O-GlcNAcylation of Orphan Nuclear Receptor Estrogen-Related Receptor gamma Promotes Hepatic Gluconeogenesis. Diabetes.

[40] Tribollet V., Barenton B., Kroiss A., Vincent S., Zhang L., Forcet C., Cerutti C., Perian S., Allioli N., Samarut J. (2016). miR-135a Inhibits the Invasion of Cancer Cells via Suppression of ERRalpha. PLoS ONE.

[41] Han L., Liu B., Jiang L., Liu J., Han S. (2016). MicroRNA-497 Downregulation Contributes to Cell Proliferation, Migration, and Invasion of Estrogen Receptor Alpha Negative Breast Cancer by Targeting Estrogen-related Receptor Alpha. Tumour Biol..

[42] Eichner L.J., Perry M.C., Dufour C.R., Bertos N., Park M., St-Pierre J., Giguère V. (2010). miR-378* Mediates Metabolic Shift in Breast Cancer Cells via the PGC-1β/ERRα Transcriptional Pathway. Cell Metab..

[43] Huss J.M., Kopp R.P., Kelly D.P. (2002). Peroxisome Proliferator-activated Receptor Coactivator-1α (PGC-1α) Coactivates the Cardiac-enriched Nuclear Receptors Estrogen-related Receptor-α and -γ. Identification of Novel Leucine-rich Interaction Motif within PGC-1α. J. Biol. Chem..

[44] Kamei Y., Ohizumi H., Fujitani Y., Nemoto T., Tanaka T., Takahashi N., Kawada T., Miyoshi M., Ezaki O., Kakizuka A. (2003). PPARgamma Coactivator 1beta/ERR Ligand 1 is an ERR Protein Ligand, Whose Expression Induces a High-energy Expenditure and Antagonizes Obesity. Proc. Natl. Acad. Sci. USA.

[45] Schreiber S.N., Knutti D., Brogli K., Uhlmann T., Kralli A. (2003). The Transcriptional Coactivator PGC-1 Regulates the Expression and Activity of the Orphan Nuclear Receptor Estrogen-related Receptor Alpha (ERRalpha). J. Biol. Chem..

[46] Laganiere J., Tremblay G.B., Dufour C.R., Giroux S., Rousseau F., Giguere V. (2004). A Polymorphic Autoregulatory Hormone Response Element in the Human Estrogen-related Receptor α (ERRα) Promoter Dictates Peroxisome Proliferator-activated Receptor α Coactivator-1α Control of ERRα Expressio. J. Biol. Chem..

[47] Sonoda J., Laganière J., Mehl I.R., Barish G.D., Chong L.W., Li X., Scheffler I.E., Mock D.C., Bataille A.R., Robert F. (2007). Nuclear Receptor ERRα and Coactivator PGC-1β are Effectors of IFN-γ Induced Host Defense. Genes Dev..

[48] Audet-Walsh E., Papadopoli D.J., Gravel S.P., Yee T., Bridon G., Caron M., Bourque G., Giguère V., St-Pierre J. (2016). The PGC-1α/ERRα Axis Represses One-carbon Metabolism and Promotes Sensitivity to Anti-folate Therapy in Breast Cancer. Cell Rep..

[49] De Vitto H., Bode A.M., Dong Z. (2019). The PGC-1/ERR Network and Its Role in Precision Oncology. NPJ Precis. Oncol..

[50] Deblois G., Giguère V. (2013). Oestrogen-related Receptors in Breast Cancer: Control of Cellular Metabolism and Beyond. Nat. Rev. Cancer.

[51] Fan W., He N., Lin C.S., Wei Z., Hah N., Waizenegger W., He M.X., Liddle C., Yu R.T., Atkins A.R. (2018). ERRgamma Promotes Angiogenesis, Mitochondrial Biogenesis, and Oxidative Remodeling in PGC1alpha/beta-Deficient Muscle. Cell Rep..

[52] Shao D., Liu Y., Liu X., Zhu L., Cui Y., Cui A., Qiao A., Kong X., Chen Q., Gupta N. (2010). PGC-1β-regulated Mitochondrial Biogenesis and Function in Myotubes is Mediated by NRF-1 and ERRα. Mitochondrion.

[53] Sonoda J., Mehl I.R., Chong L.W., Nofsinger R.R., Evans R.M. (2007). PGC-1β Controls Mitochondrial Metabolism to Modulate Circadian Activity, Adaptive Thermogenesis, and Hepatic Steatosis. Proc. Natl. Acad. Sci. USA.

[54] Vernier M., Giguère V. (2021). Aging, Senescence and Mitochondria: The PGC-1/ERR Axis. J. Mol. Endocrinol..

[55] Deblois G., St-Pierre J., Giguère V. (2013). The PGC-1/ERR Signaling Axis in Cancer. Oncogene.

[56] Bookout A.L., Jeong Y., Downes M., Yu R.T., Evans R.M., Mangelsdorf D.J. (2006). Anatomical Profiling of Nuclear Receptor Expression Reveals a Hierarchical Transcriptional Network. Cell.

[57] Huppunen J., Aarnisalo P. (2004). Dimerization Modulates the Activity of the Orphan Nuclear Receptor ERRgamma. Biochem. Biophys. Res. Commun..

[58] Audet-Walsh E., Giguère V. (2015). The Multiple Universes of Estrogen-related Receptor α and γ in Metabolic Control and Related Diseases. Acta Pharmacol. Sin..

[59] Gantner M.L., Hazen B.C., Eury E., Brown E.L., Kralli A. (2016). Complementary Roles of Estrogen-Related Receptors in Brown Adipocyte Thermogenic Function. Endocrinology.

[60] Wang T., McDonald C., Petrenko N.B., Leblanc M., Wang T., Giguère V., Evans R.M., Patel V.V., Pei L. (2015). Estrogen-Related Receptor α (ERRα) and ERRγ Are Essential Coordinators of Cardiac Metabolism and Function. Mol. Cell. Biol..

[61] Brown E.L., Hazen B.C., Eury E., Wattez J.S., Gantner M.L., Albert V., Chau S., Sanchez-Alavez M., Conti B., Kralli A. (2018). Estrogen-Related Receptors Mediate the Adaptive Response of Brown Adipose Tissue to Adrenergic Stimulation. iScience.

[62] Luo J., Sladek R., Carrier J., Bader J.-A., Richard D., Giguère V. (2003). Reduced Fat Mass in Mice Lacking Orphan Nuclear Receptor Estrogen-related Receptor α. Mol. Cell. Biol..

[63] Luo J., Sladek R., Bader J.-A., Rossant J., Giguère V. (1997). Placental Abnormalities in Mouse Embryos Lacking Orphan Nuclear Receptor ERRβ. Nature.

[64] Alaynick W.A., Kondo R.P., Xie W., He W., Dufour C.R., Downes M., Jonker J.W., Giles W., Naviaux R.K., Giguère V. (2007). ERRγ Directs and Maintains the Transition to Oxidative Metabolism in the Post-natal Heart. Cell Metab.

[65] Misra J., Kim D.K., Choi H.S. (2017). ERRγ: A Junior Orphan with a Senior Role in Metabolism. Trends Endocrinol. Metab..

[66] Alaynick W.A., Way J.M., Wilson S.A., Benson W.G., Pei L., Downes M., Yu R., Jonker J.W., Holt J.A., Rajpal D.K. (2010). ERRγ Regulates Cardiac, Gastric, and Renal Potassium Homeostasis. Mol. Endocrinol..

[67] Hatefi Y. (1985). The Mitochondrial Electron Transport and Oxidative Phosphorylation System. Annu. Rev. Biochem..

[68] Murphy M.P. (2009). How Mitochondria Produce Reactive Oxygen Species. Biochem. J..

[69] Hadrava Vanova K., Kraus M., Neuzil J., Rohlena J. (2020). Mitochondrial Complex II and Reactive Oxygen Species in Disease and Therapy. Redox Rep..

[70] Bleier L., Drose S. (2013). Superoxide Generation by Complex III: From Mechanistic Rationales to Functional Consequences. Biochim. Biophys. Acta.

[71] Charest-Marcotte A., Dufour C.R., Wilson B.J., Tremblay A.M., Eichner L.J., Arlow D.H., Mootha V.K., Giguère V. (2010). The Homeobox Protein Prox1 is a Negative Modulator of ERRα/PGC-1α Bioenergetic Functions. Genes Dev..

[72] Huss J.M., Torra I.P., Staels B., Giguère V., Kelly D.P. (2004). Estrogen-related Receptor α Directs Peroxisome Proliferator-activated Receptor α Signaling in the Transcriptional Control of Energy Metabolism in Cardiac and Skeletal Muscle. Mol. Cell. Biol..

[73] Chang C.Y., McDonnell D.P. (2012). Molecular Pathways: The Metabolic Regulator Estrogen-related Receptor Alpha as a Therapeutic Target in Cancer. Clin. Cancer Res..

[74] Schreiber S.N., Emter R., Hock M.B., Knutti D., Cardenas J., Podvinec M., Oakeley E.J., Kralli A. (2004). The Estrogen-related Receptor Alpha (ERRalpha) Functions in PPARgamma Coactivator 1alpha (PGC-1alpha)-induced Mitochondrial Biogenesis. Proc. Natl. Acad. Sci. USA.

[75] Mootha V.K., Handschin C., Arlow D., Xie X., St Pierre J., Sihag S., Yang W., Altshuler D., Puigserver P., Patterson N. (2004). Erralpha and Gabpa/b Specify PGC-1alpha-dependent Oxidative Phosphorylation Gene Expression That is Altered in Diabetic Muscle. Proc. Natl. Acad. Sci. USA.

[76] Rangwala S.M., Li X., Lindsley L., Wang X., Shaughnessy S., Daniels T.G., Szustakowski J., Nirmala N.R., Wu Z., Stevenson S.C. (2007). Estrogen-related Receptor Alpha is Essential for the Expression of Antioxidant Protection Genes and Mitochondrial Function. Biochem. Biophys. Res. Commun..

[77] Narkar V.A., Fan W., Downes M., Yu R.T., Jonker J.W., Alaynick W.A., Banayo E., Karunasiri M.S., Lorca S., Evans R.M. (2011). Exercise and PGC-1α-independent Synchronization of Type I Muscle Metabolism and Vasculature by ERRγ. Cell Metab..

[78] Hong E.-J., Levasseur M.-P., Dufour C.R., Perry M.-C., Giguère V. (2013). Loss of Estrogen-related Receptor α Promotes Hepatocellular Carcinogenesis Development via Metabolic and Inflammatory Disturbances. Proc. Natl. Acad. Sci. USA.

[79] Huss J.M., Kelly D.P. (2004). Nuclear Receptor Signaling and Cardiac Energetics. Circ. Res..

[80] Singh B.K., Sinha R.A., Tripathi M., Mendoza A., Ohba K., Sy J.A.C., Xie S.Y., Zhou J., Ho J.P., Chang C.Y. (2018). Thyroid Hormone Receptor and ERRα Coordinately Regulate Mitochondrial Fission, Mitophagy, Biogenesis, and Function. Sci. Signal..

[81] Tennessen J.M., Baker K.D., Lam G., Evans J., Thummel C.S. (2011). The Drosophila Estrogen-related Receptor Directs a Metabolic Switch That Supports Developmental Growth. Cell Metab..

[82] Beebe K., Robins M.M., Hernandez E.J., Lam G., Horner M.A., Thummel C.S. (2020). Drosophila Estrogen-related Receptor Directs a Transcriptional Switch That Supports Adult Glycolysis and Lipogenesis. Genes Dev..

[83] Panday A., Sahoo M.K., Osorio D., Batra S. (2015). NADPH Oxidases: An Overview from Structure to Innate Immunity-associated Pathologies. Cell Mol. Immunol..

[84] Rosca M.G., Vazquez E.J., Chen Q., Kerner J., Kern T.S., Hoppel C.L. (2012). Oxidation of Fatty Acids is the Source of Increased Mitochondrial Reactive Oxygen Species Production in Kidney Cortical Tubules in Early Diabetes. Diabetes.

[85] Vega R.B., Kelly D.P. (1997). A Role for Estrogen-related Receptor Alpha in the Control of Mitochondrial Fatty Acid Beta-oxidation During Brown Adipocyte Differentiation. J. Biol. Chem..

[86] Villena J.A., Hock M.B., Chang W.Y., Barcas J.E., Giguère V., Kralli A. (2007). Orphan Nuclear Receptor Estrogen-related Receptor Alpha is Essential for Adaptive Thermogenesis. Proc. Natl. Acad. Sci. USA.

[87] Birben E., Sahiner U.M., Sackesen C., Erzurum S., Kalayci O. (2012). Oxidative Stress and Antioxidant Defense. World Allergy Organ. J..

[88] Pope B.D., Ryba T., Dileep V., Yue F., Wu W., Denas O., Vera D.L., Wang Y., Hansen R.S., Canfield T.K. (2014). Topologically Associating Domains are Stable Units of Replication-timing Regulation. Nature.

[89] Yan J., Enge M., Whitington T., Dave K., Liu J., Sur I., Schmierer B., Jolma A., Kivioja T., Taipale M. (2013). Transcription Factor Binding in Human Cells Occurs in Dense Clusters Formed Around Cohesin Anchor Sites. Cell.

[90] Consortium E.P. (2012). An Integrated Encyclopedia of DNA Elements in the Human Genome. Nature.

[91] Tremblay A.M., Dufour C.R., Ghahremani M., Reudelhuber T.L., Giguère V. (2010). Physiological Genomics Identifies Estrogen-related Receptor α as a Regulator of Renal Sodium and Potassium Homeostasis and the Renin-angiotensin Pathway. Mol. Endocrinol..

[92] Saul M.C., Seward C.H., Troy J.M., Zhang H., Sloofman L.G., Lu X., Weisner P.A., Caetano-Anolles D., Sun H., Zhao S.D. (2017). Transcriptional Regulatory Dynamics Drive Coordinated Metabolic and Neural Response to Social Challenge in Mice. Genome Res..

[93] Chen X., Xu H., Yuan P., Fang F., Huss M., Vega V.B., Wong E., Orlov Y.L., Zhang W., Jiang J. (2008). Integration of External Signaling Pathways with the Core Transcriptional Network in Embryonic Stem Cells. Cell.

[94] Chronis C., Fiziev P., Papp B., Butz S., Bonora G., Sabri S., Ernst J., Plath K. (2017). Cooperative Binding of Transcription Factors Orchestrates Reprogramming. Cell.

[95] Zhao J., Lupino K., Wilkins B.J., Qiu C., Liu J., Omura Y., Allred A.L., McDonald C., Susztak K., Barish G.D. (2018). Genomic Integration of ERRgamma-HNF1beta Regulates Renal Bioenergetics and Prevents Chronic Kidney Disease. Proc. Natl. Acad. Sci. USA.

[96] Perry M.C., Dufour C.R., Tam I.S., B’Chir W., Giguère V. (2014). Estrogen-related Receptor-α Coordinates Transcriptional Programs Essential for Exercise Tolerance and Muscle Fitness. Mol. Endocrinol..

[97] Sakamoto T., Matsuura T.R., Wan S., Ryba D.M., Kim J.U., Won K.J., Lai L., Petucci C., Petrenko N., Musunuru K. (2020). A Critical Role for Estrogen-Related Receptor Signaling in Cardiac Maturation. Circ. Res..

[98] Audet-Walsh E., Yee T., McGuirk S., Vernier M., Ouellet C., St-Pierre J., Giguère V. (2017). Androgen-dependent Repression of ERRγ Reprograms Metabolism in Prostate Cancer. Cancer Res..

[99] Aquilano K., Baldelli S., Ciriolo M.R. (2014). Glutathione: New Roles in Redox Signaling for an Old Antioxidant. Front. Pharmacol..

[100] Matsuzawa A. (2017). Thioredoxin and Redox Signaling: Roles of the Thioredoxin System in Control of Cell Fate. Arch. Biochem. Biophys..

[101] Espinosa-Diez C., Miguel V., Mennerich D., Kietzmann T., Sanchez-Perez P., Cadenas S., Lamas S. (2015). Antioxidant Responses and Cellular Adjustments to Oxidative Stress. Redox Biol..

[102] Panieri E., Santoro M.M. (2016). ROS Homeostasis and Metabolism: A Dangerous Liason in Cancer Cells. Cell Death Dis..

[103] Mailloux R.J. (2018). Mitochondrial Antioxidants and the Maintenance of Cellular Hydrogen Peroxide Levels. Oxid. Med. Cell. Longev.

[104] Jezek J., Jaburek M., Zelenka J., Jezek P. (2010). Mitochondrial Phospholipase A2 Activated by Reactive oxygen Species in Heart Mitochondria Induces Mild Uncoupling. Physiol. Res..

[105] Jaburek M., Jezek J., Zelenka J., Jezek P. (2013). Antioxidant Activity by a Synergy of Redox-sensitive Mitochondrial Phospholipase A2 and Uncoupling Protein-2 in Lung and Spleen. Int. J. Biochem. Cell Biol..

[106] Jezek J., Dlaskova A., Zelenka J., Jaburek M., Jezek P. (2015). H(2)O(2)-Activated Mitochondrial Phospholipase iPLA(2)gamma Prevents Lipotoxic Oxidative Stress in Synergy with UCP2, Amplifies Signaling via G-Protein-Coupled Receptor GPR40, and Regulates Insulin Secretion in Pancreatic Beta-cells. Antioxid. Redox Signal..

[107] Olmos Y., Valle I., Borniquel S., Tierrez A., Soria E., Lamas S., Monsalve M. (2009). Mutual Dependence of Foxo3a and PGC-1alpha in the Induction of Oxidative Stress genes. J. Biol. Chem..

[108] Valle I., Alvarez-Barrientos A., Arza E., Lamas S., Monsalve M. (2005). PGC-1alpha Regulates the Mitochondrial Antioxidant Defense System in Vascular Endothelial Cells. Cardiovasc. Res..

[109] Baldelli S., Aquilano K., Ciriolo M.R. (2014). PGC-1α Buffers ROS-mediated Removal of Mitochondria During Myogenesis. Cell Death Dis..

[110] Vazquez A., Tedeschi P.M., Bertino J.R. (2013). Overexpression of the Mitochondrial Folate and Glycine-serine Pathway: A New Determinant of Methotrexate Selectivity in Tumors. Cancer Res..

[111] LeBleu V.S., O’Connell J.T., Gonzalez Herrera K.N., Wikman H., Pantel K., Haigis M.C., de Carvalho F.M., Damascena A., Domingos Chinen L.T., Rocha R.M. (2014). PGC-1α Mediates Mitochondrial Biogenesis and Oxidative Phosphorylation in Cancer Cells to Promote Metastasis. Nat. Cell Biol..

[112] Hopkins B.L., Neumann C.A. (2019). Redoxins as Gatekeepers of the Transcriptional Oxidative Stress Response. Redox Biol..

[113] Holmstrom K.M., Baird L., Zhang Y., Hargreaves I., Chalasani A., Land J.M., Stanyer L., Yamamoto M., Dinkova-Kostova A.T., Abramov A.Y. (2013). Nrf2 Impacts Cellular Bioenergetics by Controlling Substrate Availability for Mitochondrial Respiration. Biol. Open.

[114] Carter E.L., Ragsdale S.W. (2014). Modulation of Nuclear Receptor Function by Cellular Redox Poise. J. Inorg. Biochem..

[115] Yang W., Hekimi S. (2010). A Mitochondrial Superoxide Signal Triggers Increased Longevity in Caenorhabditis Elegans. PLoS Biol..

[116] Cox C.S., McKay S.E., Holmbeck M.A., Christian B.E., Scortea A.C., Tsay A.J., Newman L.E., Shadel G.S. (2018). Mitohormesis in Mice via Sustained Basal Activation of Mitochondrial and Antioxidant Signaling. Cell Metab..

[117] Herzig S., Shaw R.J. (2018). AMPK: Guardian of Metabolism and Mitochondrial Homeostasis. Nat. Rev. Mol. Cell Biol..

[118] Kang S.W., Haydar G., Taniane C., Farrell G., Arias I.M., Lippincott-Schwartz J., Fu D. (2016). AMPK Activation Prevents and Reverses Drug-Induced Mitochondrial and Hepatocyte Injury by Promoting Mitochondrial Fusion and Function. PLoS ONE.

[119] Jezek J., Cooper K.F., Strich R. (2018). Reactive Oxygen Species and Mitochondrial Dynamics: The Yin and Yang of Mitochondrial Dysfunction and Cancer Progression. Antioxidants.

[120] Willems P.H., Rossignol R., Dieteren C.E., Murphy M.P., Koopman W.J. (2015). Redox Homeostasis and Mitochondrial Dynamics. Cell Metab..

[121] Hales K.G., Fuller M.T. (1997). Developmentally Regulated Mitochondrial Fusion Mediated by a Conserved, Novel, Predicted GTPase. Cell.

[122] Santel A., Fuller M.T. (2001). Control of Mitochondrial Morphology by a Human Mitofusin. J. Cell Sci..

[123] Delettre C., Lenaers G., Griffoin J.M., Gigarel N., Lorenzo C., Belenguer P., Pelloquin L., Grosgeorge J., Turc-Carel C., Perret E. (2000). Nuclear Gene OPA1, Encoding a Mitochondrial Dynamin-related Protein, is Mutated in Dominant Optic Atrophy. Nat. Genet..

[124] Cartoni R., Leger B., Hock M.B., Praz M., Crettenand A., Pich S., Ziltener J.L., Luthi F., Deriaz O., Zorzano A. (2005). Mitofusins 1/2 and ERRalpha Expression are Increased in Human Skeletal Muscle After Physical Exercise. J. Physiol..

[125] Soriano F.X., Liesa M., Bach D., Chan D.C., Palacin M., Zorzano A. (2006). Evidence for a Mitochondrial Regulatory Pathway Defined by Peroxisome Proliferator-activated Receptor-gamma Coactivator-1 Alpha, Estrogen-related Receptor-alpha, and Mitofusin 2. Diabetes.

[126] Tsushida K., Tanabe K., Masuda K., Tanimura S., Miyake H., Arata Y., Sugiyama H., Wada J. (2018). Estrogen-related Receptor α is Essential for Maintaining Mitochondrial Integrity in Cisplatin-induced Acute Kidney Injury. Biochem. Biophys. Res. Commun..

[127] Lee J.E., Westrate L.M., Wu H., Page C., Voeltz G.K. (2016). Multiple Dynamin Family Members Collaborate to Drive Mitochondrial Division. Nature.

[128] Smirnova E., Griparic L., Shurland D.L., van der Bliek A.M. (2001). Dynamin-related Protein Drp1 is Required for Mitochondrial Division in Mammalian Cells. Mol. Biol. Cell.

[129] Gong W., Song J., Chen X., Li S., Yu J., Xia W., Ding G., Zhang Y., Jia Z., Zhang A. (2019). Estrogen-related Receptor-alpha Mediates Puromycin Aminonucleoside-induced Mesangial Cell Apoptosis and Inflammatory Injury. Am. J. Physiol. Renal. Physiol..

[130] Wang M., Yang G., Jiang X., Lu D., Mei H., Chen B. (2017). Peroxisome Proliferator-Activated Receptor-gamma Coactivator-1alpha (PGC-1alpha) Regulates the Expression of B-Cell Lymphoma/Leukemia-2 (Bcl-2) and Promotes the Survival of Mesenchymal Stem Cells (MSCs) via PGC-1alpha/ERRalpha Interaction in the Absence of Serum, Hypoxia, and High Glucose Conditions. Med. Sci. Monit..

[131] Huang M., Chen L., Mao X., Liu G., Gao Y., You X., Gao M., Sehouli J., Sun P. (2020). ERRalpha Inhibitor Acts as a Potential Agonist of PPARgamma to Induce Cell Apoptosis and Inhibit Cell Proliferation in Endometrial Cancer. Aging.

[132] Loboda A., Jozkowicz A., Dulak J. (2010). HIF-1 and HIF-2 Transcription Factors--similar but not Identical. Mol. Cells.

[133] Ao A., Wang H., Kamarajugadda S., Lu J. (2008). Involvement of Estrogen-related Receptors in Transcriptional Response to Hypoxia and Growth of Solid Tumors. Proc. Natl. Acad. Sci. USA.

[134] Zou C., Yu S., Xu Z., Wu D., Ng C.F., Yao X., Yew D.T., Vanacker J.M., Chan F.L. (2014). ERRalpha Augments HIF-1 Signalling by Directly Interacting with HIF-1alpha in Normoxic and Hypoxic Prostate Cancer Cells. J. Pathol..

[135] Li Y., Padmanabha D., Gentile L.B., Dumur C.I., Beckstead R.B., Baker K.D. (2013). HIF- and Non-HIF-regulated Hypoxic Responses Require the Estrogen-related Receptor in Drosophila Melanogaster. PLoS Genet..

[136] Yuk J.M., Kim T.S., Kim S.Y., Lee H.M., Han J., Dufour C.R., Kim J.K., Jin H.S., Yang C.S., Park K.S. (2015). Orphan Nuclear Receptor ERRα Controls Macrophage Metabolic Signaling and A20 Expression to Negatively Regulate TLR-Induced Inflammation. Immunity.

[137] Hamidian A., von Stedingk K., Munksgaard Thoren M., Mohlin S., Pahlman S. (2015). Differential Regulation of HIF-1alpha and HIF-2alpha in Neuroblastoma: Estrogen-related Receptor Alpha (ERRalpha) Regulates HIF2A Transcription and Correlates to Poor Outcome. Biochem. Biophys. Res. Commun..

[138] Rasbach K.A., Gupta R.K., Ruas J.L., Wu J., Naseri E., Estall J.L., Spiegelman B.M. (2010). PGC-1alpha Regulates a HIF2alpha-dependent Switch in Skeletal Muscle Fiber Types. Proc. Natl. Acad. Sci. USA.

[139] Kim J.H., Choi Y.K., Byun J.K., Kim M.K., Kang Y.N., Kim S.H., Lee S., Jang B.K., Park K.G. (2016). Estrogen-related Receptor Gamma is Upregulated in Liver Cancer and Its Inhibition Suppresses Liver Cancer Cell Proliferation via Induction of p21 and p27. Exp. Mol. Med..

[140] Kim S.Y., Yang C.S., Lee H.M., Kim J.K., Kim Y.S., Kim Y.R., Kim J.S., Kim T.S., Yuk J.M., Dufour C.R. (2018). ESRRA (Estrogen-related Receptor α) is a Key Coordinator of Transcriptional and Post-translational Activation of Autophagy to Promote Innate Host Defense. Autophagy.

[141] Murray J., Auwerx J., Huss J.M. (2013). Impaired Myogenesis in Estrogen-related Receptor Gamma (ERRgamma)-deficient Skeletal Myocytes Due to Oxidative Stress. FASEB J..

[142] Marin-Royo G., Rodriguez C., Le Pape A., Jurado-Lopez R., Luaces M., Antequera A., Martinez-Gonzalez J., Souza-Neto F.V., Nieto M.L., Martinez-Martinez E. (2019). The Role of Mitochondrial Oxidative Stress in the Metabolic Alterations in Diet-induced Obesity in Rats. FASEB J..

[143] Houstis N., Rosen E.D., Lander E.S. (2006). Reactive Oxygen Species Have a Causal Role in Multiple Forms of Insulin Resistance. Nature.

[144] Dufour C.R., Levasseur M.-P., Pham N.H.H., Eichner L.J., Wilson B.J., Charest-Marcotte A., Duguay D., Poirier-Héon J.-F., Cermakian N., Giguère V. (2011). Genomic Convergence Among ERRα, Prox1 and Bmal1 in the Control of Metabolic Clock Outputs. PLoS Genet..

